# Manipulation of Metabolic Pathways to Develop Vitamin-Enriched Crops for Human Health

**DOI:** 10.3389/fpls.2017.00937

**Published:** 2017-06-06

**Authors:** Ling Jiang, Weixuan Wang, Tong Lian, Chunyi Zhang

**Affiliations:** ^1^Biotechnology Research Institute, Chinese Academy of Agricultural SciencesBeijing, China; ^2^National Key Facility for Crop Gene Resources and Genetic ImprovementBeijing, China

**Keywords:** vitamins, metabolism, biofortification, crop, human health

## Abstract

Vitamin deficiencies are major forms of micronutrient deficiencies, and are associated with huge economic losses as well as severe physical and intellectual damages to humans. Much evidence has demonstrated that biofortification plays an important role in combating vitamin deficiencies due to its economical and effective delivery of nutrients to populations in need. Biofortification enables food plants to be enriched with vitamins through conventional breeding and/or biotechnology. Here, we focus on the progress in the manipulation of the vitamin metabolism, an essential part of biofortification, by the genetic modification or by the marker-assisted selection to understand mechanisms underlying metabolic improvement in food plants. We also propose to integrate new breeding technologies with metabolic pathway modification to facilitate biofortification in food plants and, thereby, to benefit human health.

## Introduction

Vitamins are organic compounds required by human as micronutrients in trace amounts.^1,2^ Adequate quantity of vitamins refers to the correct quantity of vitamins from food that is actually required by human body. People use the RDI to describe the quantity to meet the requirements of 97–98% of healthy individuals for the prevention of clinical deficiency. The RDI is a whole set of the daily intake level of a nutrient that is considered to be sufficient for people. Vitamin deficiencies are major forms of micronutrient deficiencies, due to insufficient intake of vitamins, threatening billion of people, and causing nutrition-related poor growth (FAO, 2009). For example, vitamin A deficiency results in night blindness, xerophthalmia, measles, corneal scarring and death of children; and folate deficiency in pregnant females frequently results in neural tube defects in new-borns (FAO/WHO, 2001).

Biofortification, a type of micronutrient intervention, aims to increase micronutrients in seeds, tubers, and leafy vegetables of food crops, and has the potential to reach the rural poor more effectively, the group who are often at highest risk of micronutrient deficiencies (Hotz and McClafferty, 2007). Nutritionists, biologists and breeders have, for decades, focused on promoting the bioavailability of micronutrients in edible parts of crops (Hurrell and Egli, 2010). In this review, we give a special attention to the progress in the manipulation of the vitamin metabolism for developing vitamin-enriched crops.

## Manipulation of Vitamin Metabolism

### Provitamin A

**FIGURE 1:**
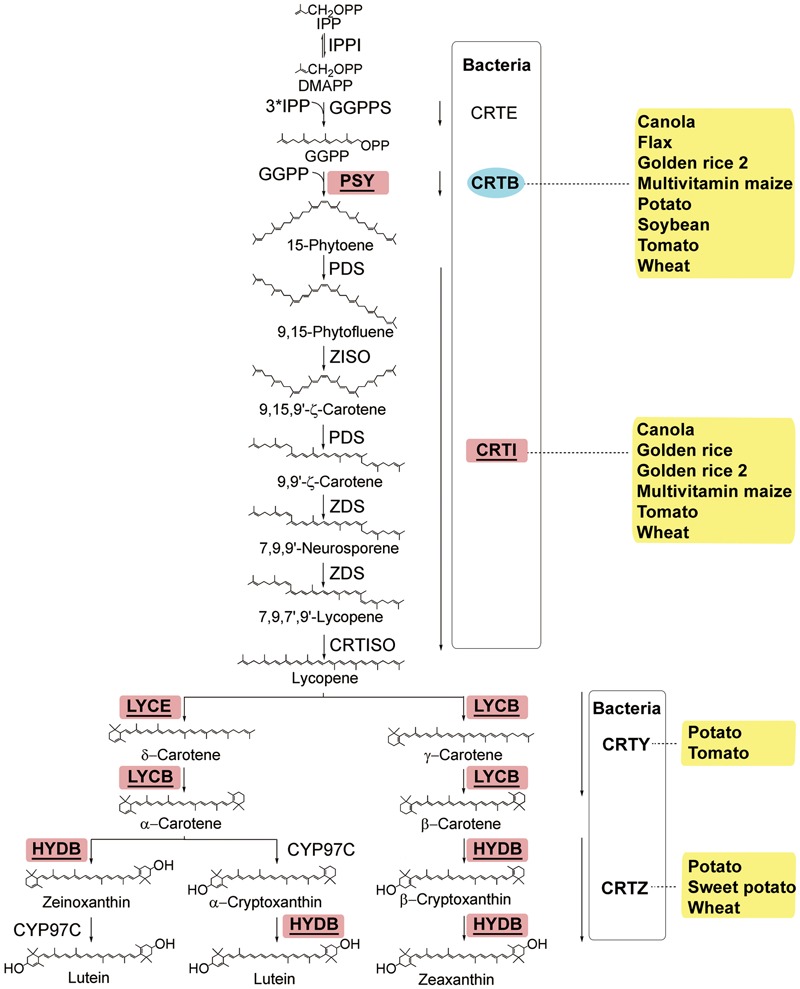
The carotenoid biosynthesis pathway in plants and equivalent steps in bacteria (modified from Bai et al., 2011). Enzymes: CRTB, bacterial phytoene synthase; CRTE, bacterial GGPP synthase; CRTI, bacterial phytoene desaturase/isomerase; CRTISO, carotenoid isomerase; CRTY, bacterial lycopene cyclase; CRTZ, bacterial β-carotene hydroxylase; CYP97C, carotene 𝜀-ring hydroxylase; GGPPS, GGPP synthase; HYDB, β-carotene hydroxylase; IPPI, isopentenyl diphosphate isomerase; LYCB, lycopene β-cyclase; LYCE, lycopene 𝜀-cyclase; PDS, phytoene desaturase; PSY, phytoene synthase; ZDS, ζ-carotene desaturase; Z-ISO, ζ-carotene isomerase. Chemicals: DMAPP, dimethylallyl diphosphate; GGPP, geranylgeranyl diphosphate; IPP, isopentenyl diphosphate. The bacterial pathway is put in a box with the hard line. The transgenic species are indicated adjacent to enzymes used in manipulation. Enzymes used only in transgenic strategies are oval and in the blue background, and enzymes used in both transgenic strategies and genetic breeding are square, underlined, and in the red background.

Vitamin A exists in several forms known as retinoids. Human can synthesize retinal from the abundant provitamin A carotenoids present in fruits and vegetables such as oranges (*Citrus aurantium*), broccoli (*Brassica oleracea*), spinach (*Spinacia oleracea*), carrot (*Daucus carota*), squash (*Cucurbita maxima*), sweet potato (*Ipomoea batatas*), and pumpkin (*Cucurbita maxima*) (Harrison, 2005). Plants produce four types of provitamin A carotenoids from isopentenyl diphosphate (IPP), which can form the intermediate product phytoene. Among carotenoids, α-carotene and β-carotene accumulate in greater amounts than γ-carotene and β-cryptoxanthin, which tend to be converted rapidly into downstream products in several forms known as retinoids (reviewed by Bai et al., 2011; **Figure 1**).

The RDI of vitamin A is 700 μg retinol equivalents per day^2^; however, provitamin A is non-detectable in rice (*Oryza sativa*), millet (*Panicum miliaceum*), and sorghum (*Sorghum bicolor*), and low in wheat (*Triticum aestivum*), barley (*Hordeum vulgare*), and potato (*Solanum tuberosum*)^1^. In plants, β-carotene yields β-cryptoxanthin, which is further converted into zeaxanthin, whereas α-carotene yields lutein; and these chemicals are non-provitamin carotenoids (**Figure 1**). Both conventional and biotechnological approaches have been used to enhance the biosynthesis of provitamin A carotenoids in crops. The most commonly used gene for carotenoids biofortification is the gene encoding a phytoene synthase (PSY in plant; CRTB in bacteria; **Figure 1**). In Golden Rice, the introduction of a *Pantoea ananatis* phytoene desaturase (*CrtI*) and a *Narcissus pseudonarcissus* phytoene synthase (*PSY*) into rice results in an accumulation of 1.6 μg total carotenoids per gram of dry seeds (Ye et al., 2000). In Golden Rice 2, a maximum of 37 μg g^-1^ total carotenoids with β-carotene preferentially accumulated in the endosperm is achieved when a maize (*Zea mays*) *PSY1* gene is utilized (Paine et al., 2005). In these two events, the enhanced phytoene synthesis guarantees an adequate metabolic flux throughout the pathway. Similar strategies, i.e., overexpression of the phytoene synthase gene alone, phytoene desaturase gene alone, or combination of these two genes, are applied to other crops including canola (*Brassica napus*; both genes in Ravanello et al., 2003; Aluru et al., 2008; *crtB* in Shewmaker et al., 1999), flax (*Linum usitatissimum*; *crtB* in Fujisawa et al., 2008), potato (*crtB* in Ducreux et al., 2005), tomato (*Solanum lycopersicum*; *crtB* in Fraser et al., 2002; both genes in Aluru et al., 2008), maize (both genes in Naqvi et al., 2009), soybean (*Glycine max*; *crtB* in Schmidt et al., 2015), and wheat (both genes in Wang et al., 2014), among which the highest fold change of β-carotene is observed in soybean (**Figure 1**). A chimeric gene consisting of a chloroplast signal from pea (*Pisum sativum*) and a *crtB* from bacterium *Pantoea* is introduced into soybean via biolistics, resulting in an accumulation of 845 μg/g β-carotene, about 1500-fold increase as compared to wild type, in dry seeds (Schmidt et al., 2015).

Some other approaches are also used to enhance carotenoid biosynthesis in plants. Lycopene β-cyclase (LYCB), lycopene 𝜀-cyclase (LYCE), and HYDB are key enzymes in the β-branch of the carotenoid biosynthesis pathway. To achieve a high level of β-carotene accumulation, suppressing the activity of LYCE by silencing *StLCY-e* in potato or the activity of HYDB by silencing *StCHY-𝜀* in potato is adopted to ensure the appropriate direction of the metabolic flux (Diretto et al., 2007; Van Eck et al., 2007). In wheat, simultaneous overexpression of *CrtB* and silencing of *HYDB* achieves an up to 31-fold increase of β-carotene up to 5.06 μg g^-1^ (Zeng et al., 2015). Another approach is to make use of the *Orange* (*Or*) gene from cauliflower (*Brassica oleracea* var. *botrytis*, Lu et al., 2006). The OR protein is responsible for the accumulation of carotenoids in plants, and interacts with PSY to increase the stability and activity at a post-translational level for carotenoid biosynthesis (Zhou et al., 2015). Transgenic potato tubers expressing *Or* exhibit elevated carotenoids, and the β-carotene is increased continuously during long-term cold storage (Lopez et al., 2008; Li L. et al., 2012). Similar strategy is successful in tomato (Bai et al., 2014).

However, these strategies can also have disadvantages in some cases. For example, overexpression of *PSY* in tomato increases lycopene, β-carotene, and zeaxanthin, but decreases gibberellins, which results in plant dwarfism (Fray et al., 1995). This observation indicates that manipulation of the provitamin A metabolism is still limited by our incomplete understanding of the regulation of the endogenous pathways, including rate-controlling steps and timing of expression in carotenogenic tissues (Vallabhaneni and Wurtzel, 2009). Therefore, identification of the allelic variations that control carotenoid biosynthesis by means of candidate gene-based genome-wide association studies (GWASs) to better facilitate provitamin A biofortification through allele pyramiding has become an important alternative. Much work to identify the genes or QTLs responsible for the carotenoid accumulation has been done in maize, given its marked genetic diversity (Palaisa et al., 2003; Vallabhaneni and Wurtzel, 2009; Yan et al., 2010; Azmach et al., 2013; Owens et al., 2014; **Figure 1**). Besides, maize lines containing such natural variations can be used as donor parents to accelerate developing provitamin A-fortified tropical maize varieties, adapted to target growth conditions and consumer preferences. For example, the β-carotene-rich hybrid maize has developed through the marker-assisted introgression of the natural alleles in β-carotene hydroxylase gene by using a crtRB1-specific DNA marker for foreground selection. (Muthusamy et al., 2014).

To identify other metabolic bottlenecks in the carotenoid pathway, the genes of *Arabidopsis* 1-deoxy-D-xylulose-5-phosphate synthase (AtDXS) and *Arabidopsis* ORANGE (AtOR) are introduced into Golden Rice 2. Upon the transformation of *AtDXS*, accumulation of the carotenoids in the endosperm is significantly enhanced, confirming that the supply of isoprenoid precursors such as geranylgeranyl diphosphate (GGPP) is a rate-limiting step (Bai et al., 2016). In Sorghum, AtDXS was coexpressed with PSY1 and CRTI, and the transgenic events accumulated higher levels of total carotenoids, and the all-trans β-carotene levels ranged from 2.5 to 9.1 μg g^-1^ DW in the mature-seed endosperm (Che et al., 2016). Upon the transformation of *AtOR*, levels of the carotenoids are also dramatically elevated, indicating that OR functions mainly through expanding the metabolic sink (Bai et al., 2016). Obviously, identification of the metabolic bottlenecks in the carotenoid pathway can help to refine the strategies for development of crops with specific carotenoids.

### Folates

**FIGURE 2:**
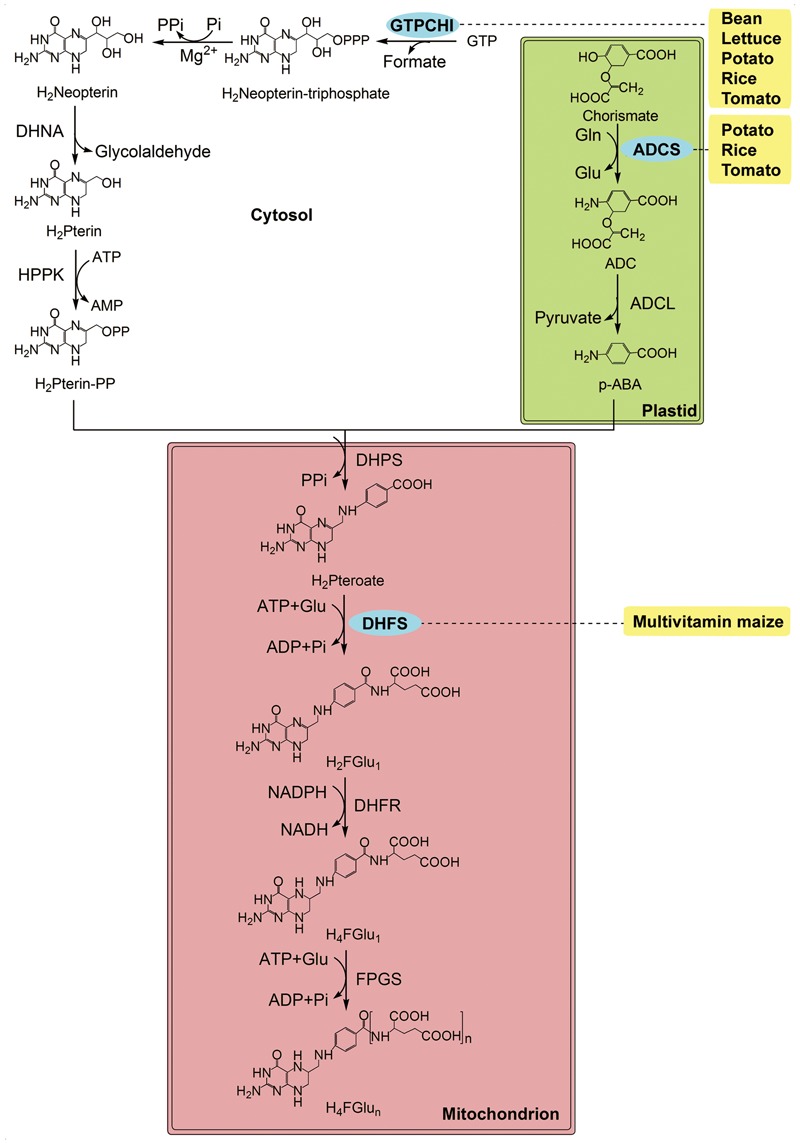
The folate biosynthesis pathway and its compartmentalisation in plants (modified from Storozhenko et al., 2005). p-ABA is synthesized in the plastids, pteridine in the cytosol and both are condensed to form THF in the mitochondria. Enzymes: ADCL, Aminodeoxychorismate lyase; ADCS, Aminodeoxychorismate synthase; DHFR, dihydrofolate reductase; DHFS, dihydrofolate synthetase; DHNA, dihydroneopterin aldolase; DHPS, dihydropteroate synthase; FPGS, folylpolyglutamate synthase; GTPCHI, GTP cyclohydrolase I; HPPK, dihydropterinpyrophosphokinase. Chemicals: ADC, minodeoxychorismate; H_2_FGlu_1_, dihydrofolate; H_2_Neopterin, dihydroneopterin; H_2_Pteroate, dihydropteroate; Glu, glutamate; GTP, guanosine triphosphate; H_2_Pterin, dihydropterin; H_2_Pterin-PP, hydroxymethyldihydropterin; p-ABA, para-aminobenzoate; H_4_FGlu_1_, tetrahydrofolate. The transgenic species are indicated adjacent to enzymes used in manipulation. Enzymes used in transgenic strategies are oval and in the blue background.

Folates, also called vitamin B_9_, are essential water-soluble B-vitamins, including tetrahydrofolate (THF) and its derivatives. Folates play an important role as one-carbon donors and acceptors in all organisms. Folates are synthesized *de novo* in bacteria, fungi and plants. It is noteworthy that the folate biosynthesis pathway is split among cytosol, mitochondria, and chloroplasts in plants, whereas it is cytosolic in other organisms (reviewed by Storozhenko et al., 2005; Blancquaert et al., 2010, **Figure 2**). Usually, folates are abundant in dark-green leafy vegetables, but only tens of micrograms per 100 g weight in barley, corn, lettuce (*Lactuca sativa*), potato, rice, sweet potato, tomato, wheat, etc^1^. Therefore, folate intake is prone to inadequacy, i.e., less than the RDI of 400 μg per day for adults^2^, if the dietary pattern is not optimized.

Scientists have made lots of efforts to develop various folate-fortified crops using biotechnology in recent years, including tomato, rice, maize, lettuce, potato, and Mexican common bean (*Phaseolus vulgaris*) (Diaz de la Garza et al., 2004, 2007; Storozhenko et al., 2007; Naqvi et al., 2009; Nunes et al., 2009; Blancquaert et al., 2013; Ramirez Rivera et al., 2016; **Figure 2**). In general, two approaches for folate biofortification have been employed. One is to overexpress dihydrofolate synthetase (DHFS; FOLE in bacteria), a rate-limiting enzyme in folate synthesis, achieving an only 2-fold increase in *folE*-overexpressing corn endosperm up to 1.94 μg g^-1^ (Naqvi et al., 2009). The other strategy, i.e., overexpression of the enzymes catalyzing the first committed step in the cytosol (CTPCHI), increases folates by 2 folds in tomato up to 2.99 nmol g^-1^(Diaz de la Garza et al., 2004), 8.5 folds in lettuce up to 1.85 μg g^-1^ (Nunes et al., 2009), respectively. Similar strategy is used in Mexican common bean and increased pteridine by 3 folds up to 3.25 μg g^-1^ (Ramirez Rivera et al., 2016). A greater increase in tomato (25 folds, up to 8.40 μg g^-1^) and rice (100 folds, up to 17.23 μg g^-1^) is achieved by crossing the pteridine-overproducing traits with para-aminobenzoate (p-ABA)-overproducing traits (Diaz de la Garza et al., 2007; Storozhenko et al., 2007).

A similar strategy does not work in potato, regardless of the high expression of *GTPCHI* and *Aminodeoxychorismate synthase* (*ADCS*): folates in the immature potato tubers are mildly increased (∼ 2 folds, up to 1.23 μg g^-1^), and expression of the endogenous *GTPCHI* and *ADCS* does not differ between the WT and transgenic lines (Blancquaert et al., 2013). These observations are markedly different from those reported in tomato and rice (Storozhenko et al., 2007; Waller et al., 2010). Therefore, probably there is a bottleneck in the folate biosynthesis pathway in potato tubers, and a further research is needed to investigate the mechanisms of regulating folate status in these plants that are different, lacking, or of minor importance in rice seeds and tomato fruit. Besides, the combination of folylpolyglutamate synthetase (FPGS), GTPCHI, and ADCS from *Arabidopsis* with folate binding proteins (FBP) from mammals has been successful in improving folate accumulation (up to 25.30 μg g^-1^) and stability (folate levels were stable for 4 months at 28°C) in rice (Blancquaert et al., 2015). These results indicate that a better understanding of the folate pathway is required to formulate an engineering strategy useful for the majority of other staple crops (Blancquaert et al., 2013).

### Other B-class Vitamins

B-class vitamins, other than folates, include VB_1_ (thiamine), VB_2_ (riboflavin), VB_3_ (niacin), VB_5_ (pantothenic acid), VB_6_ (pyridoxal, pyridoxine, pyridoxamine, and their phosphorylated derivatives), VB_7_ (biotin), and VB_12_ (cobalamin). To our knowledge, however, only VB_6_ metabolic engineering has been mainly conducted in cassava (*Manihot esculenta*; Vanderschuren et al., 2013; Li et al., 2015).

**FIGURE 3:**
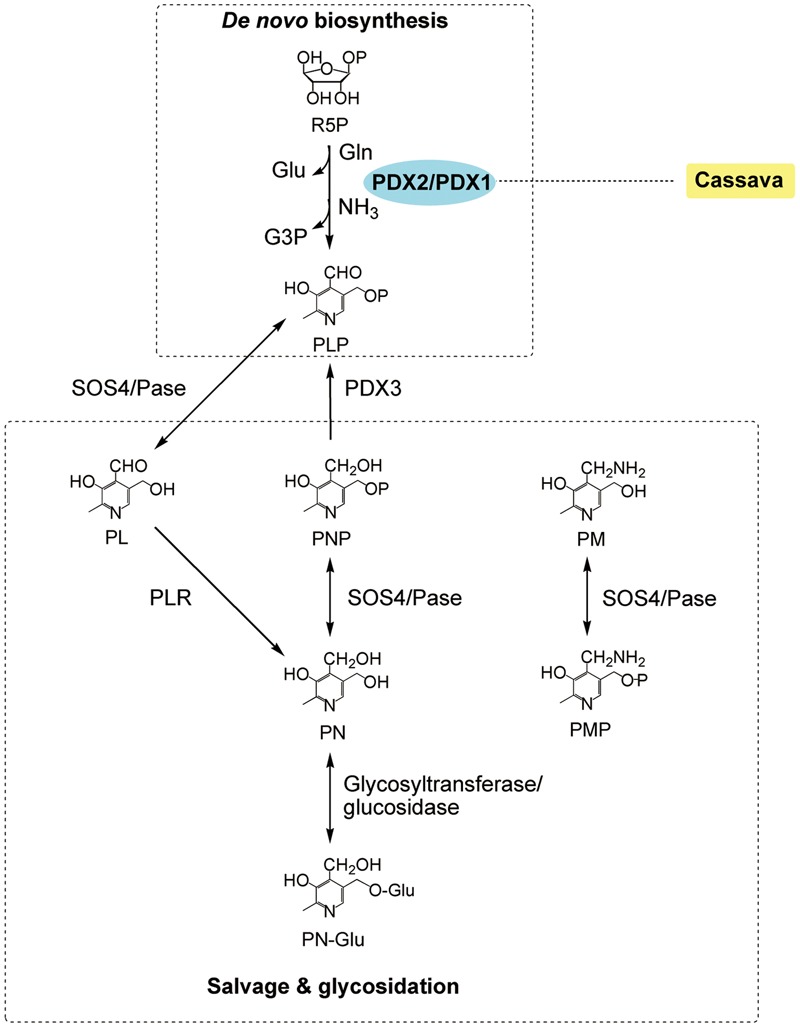
The VB_6_ metabolism in plants (modified from Leuendorf et al., 2010). The VB_6_ metabolism includes *de no* biosynthesis, salvage glycosidation. Enzymes: PDX1, pyridoxal-5′-phosphate synthase; PDX2, pyridoxal-5′-phosphate glutaminase; PDX3, pyridoxine-5′-phosphate/pyridoxamine-5′-phosphate oxidase; PLR, pyridoxal reductase; SOS4/Pase, pyridoxine/pyridoxal/pyridoxamine kinase. Chemicals: Gln, glutamine; Glu, glutamate; G3P, glyceraldehydes 3-phosphate; PA, pyridoxic acid; PN, pyridoxine; PN-Glu, PN-glycoside; PL, pyridoxal; PLP, pyridoxal-5′-phosphate; PM, pyridoxamine; PMP, pyridoxamine-5′-phosphate; PNP, pyridoxine-5′-phosphate; R5P, ribose 5-phosphate. The transgenic species are indicated adjacent to enzymes used in manipulation. Enzymes used in transgenic strategies are oval and in the blue background.

VB_6_ refers to a group of six water-soluble vitamers, among which pyridoxal-5′-phosphate (PLP) is of central importance because it is required as a cofactor for over 140 chemical reactions in the cell (Vanderschuren et al., 2013; **Figure 3**). The RDI for vitamin VB_6_ is 1.3 mg per day for adults^2^. Several groups reported overexpression of the genes either encoding pyridoxal phosphate synthase (PDX1) or encoding pyridoxal phosphate glutaminase (PDX2) in *Arabidopsis*, but the increase of VB_6_ is not significant in most cases (Chen and Xiong, 2009; Leuendorf et al., 2010; Raschke et al., 2011). Later on, however, the simultaneous overexpression of *Arabidopsis PDX1.1* and *PDX2* genes in cassava achieved a 9.0-fold increase in the leaves to 54.74 μg g^-1^ by *CaMV35S* promoter, and a 15.4-fold increase in the roots to 16.21 μg g^-1^ by root-enhanced *Patatin* promoter, respectively (Li et al., 2015). Apparently, understanding of the regulatory mechanisms underlying the metabolic pathways can be improved in order for people to eventually benefit from the biofortification.

### Vitamin C

**FIGURE 4:**
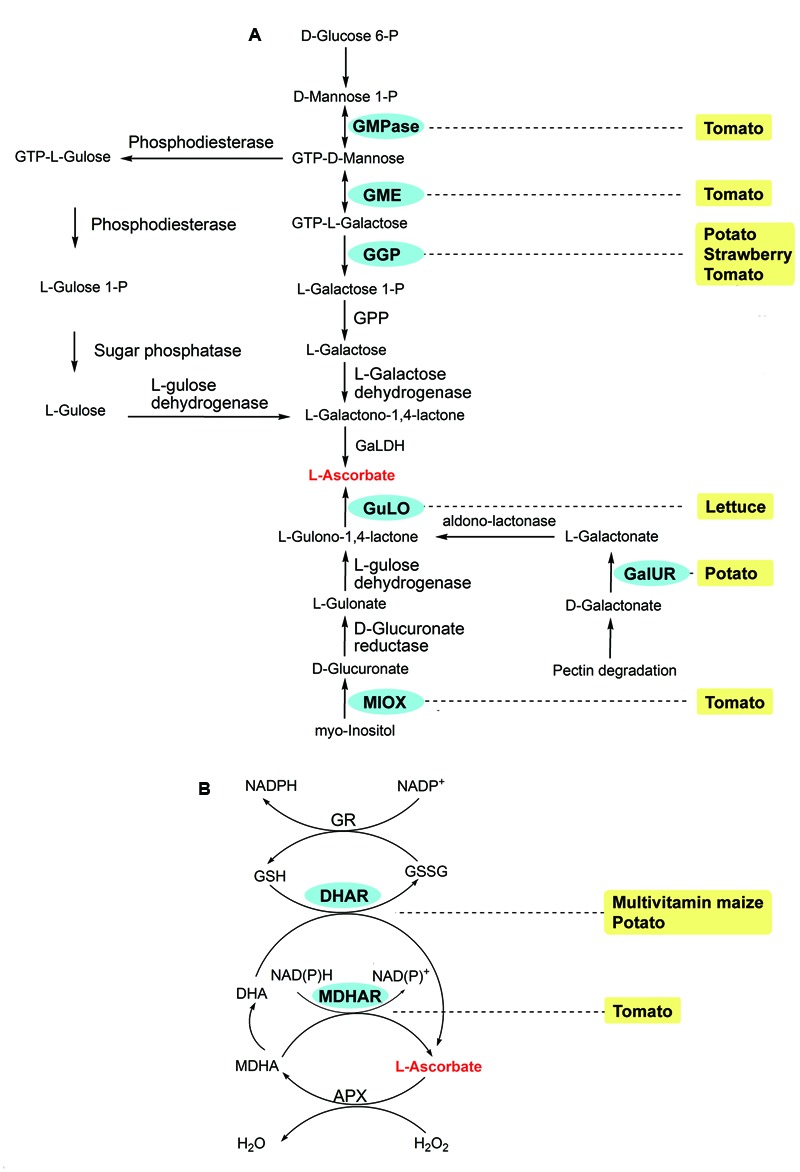
ASC biosynthetic routes **(A)** and Foyer-Halliwell-Asada cycle **(B)**, also known as the ASC-GSH cycle) in plants (modified from Locato et al., 2013). Enzymes: APX, ascorbateperoxidase; DHAR, dehydroascorbate reductase; GaIUR, Galacturonate reductase; GGP, GDP-Galactose phosphorylase; GME, GDP-Mannose epimerase; GMPase, GDP-Mannose pyrophosphorylase; GPP, L-Galactose 1P phosphatase; GaLDH, L-Galactono-1,4-γ-lactone dehydrogenase; GR, glutathione reductase; GuLO, L-Gulono-1,4-γ-lactone oxidase; MDHAR, monodehydroascorbate reductase; MIOX, Myo-inositol oxygenase. Chemicals: DHA, dehydroascorbate; GSH, glutathione; GSSG, oxidized glutathione; MDHA, monodehydroascorbate. The transgenic species are indicated adjacent to enzymes used in manipulation. Enzymes used in transgenic strategies are oval and in the blue background.

Vitamin C, also known as ascorbate, can be synthesized via four pathways. In plants, D-glucose 6-P, D-galacturonate, and myo-inositol are the substrates of the Smirnoff-Wheeler, pectin degradation, animal, and animal-like pathways, respectively (reviewed by Locato et al., 2013; **Figure 4A**). The RDI for vitamin C is 75 mg per day for adults^2^. Potato and tomato contain one fourth to one fifth of the RDI for vitamin C per 100 g weight; while vitamin C is quite low and usually undetectable in the grains of cereal crops, including barley, corn, millet, rice, and wheat^1^.

Vitamin C biofortification has been carried out in lettuce (Jain and Nessler, 2000), maize (Chen et al., 2003; Naqvi et al., 2009), potato (Hemavathi et al., 2009; Qin et al., 2011; Zhang et al., 2011; Bulley et al., 2012), tomato (Bulley et al., 2012; Cronje et al., 2012; Gest et al., 2013), and strawberry (*Fragaria ananassa*; Bulley et al., 2012) by either overexpressing the genes involved in biosynthesis or silencing the genes involved in ascorbate recycling (**Figure 4**). Most of the overexpression in *Arabidopsis*, tomato, strawberry and potato results in a 2- to 6-fold increase in ascorbate (Lorence et al., 2004; Hemavathi et al., 2009; Zhang et al., 2011; Bulley et al., 2012; Cronje et al., 2012). A 7-fold increase of ascorbate, the largest increase observed up to date, was achieved in transgenic lettuce when a rat L-Gulono-1,4-γ-lactone oxidase (GuLO) was overexpressed (Jain and Nessler, 2000). Unlike the overexpression, knockdown of the genes participating in recycling, such as monodehydroascorbate reductase (MDHAR) and dehydroascorbate reductase (DHAR), leads to less increase in tomato, maize, and potato (Chen et al., 2003; Naqvi et al., 2009; Qin et al., 2011; Gest et al., 2013; **Figure 4B**).

### Vitamin E

**FIGURE 5:**
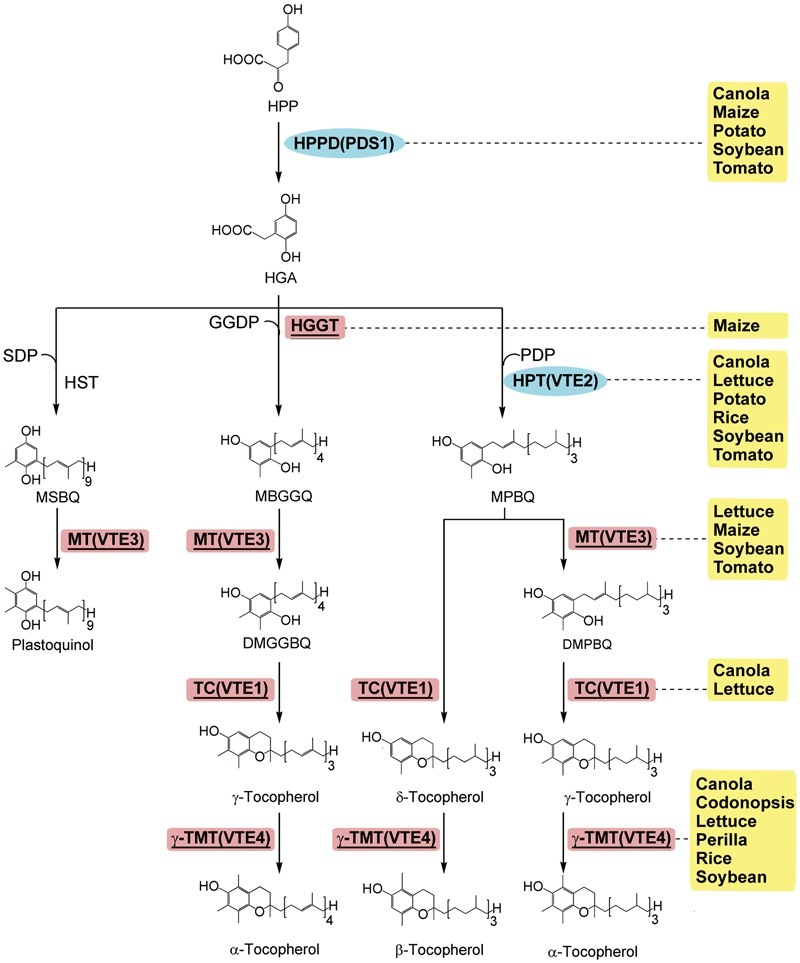
The enzymatic steps and metabolic products of tocopherol synthesis in plants (modified from Dörmann, 2007). Enzymes: HGGT, homogentisate geranylgeranyl transferase; HPPD/PDS1, p-Hydroxyphenylpyruvic acid dioxygenase; HPT/VTE2, homogentisic acid prenyltransferase; HST, homogentisate solanesyltransferase; MT/VTE3, MPBQ methyltransferase; TC/VTE1, tocopherol cyclase; γ-TMT/VTE4, γ-tocopherol methyltransferase. Chemicals: DMBPQ, 2,3-dimethyl-5-phytyl-benzoquinol; DMGGBQ, 2,3-dimethyl-5-geranylgeranylbenzoquinol; GGDP, geranylgeranyl-diphosphate; HGA, homogentisic acid; MEP, 2-*C*-methyl-D-erythritol 4-phosphate; MGGBQ, 2-methyl-6-geranylgeranyl-benzoquinol; MPBQ, 2-methyl-6-phytyl-1,4-benzoquinol; MSBQ, 2-methyl-6-solanesyl-benzoquinol; PDP, phytyldiphosphate; SDP, solanesyl-diphsophate. The transgenic species are indicated adjacent to enzymes used in manipulation. Enzymes used only in transgenic strategies are oval and in the blue background, and enzymes used in both transgenic strategies and genetic breeding are square, underlined, and in the red background.

Vitamin E is important for human health, and dietary or supplemental vitamin E is absorbed and delivered to the liver (Traber, 2007). Plants are the primary source of dietary vitamin E, producing the tocopherol and tocotrienol derivatives that collectively constitute vitamin E (Chen et al., 2006). The RDI for vitamin E is 15 mg per day for adults^1,2^. Tocotrienols are the major form of vitamin E in the seeds of most monocots and a limited number of dicots (Cahoon et al., 2003). Tocopherols have one saturated phytyl tail, while tocotrienols have 3-fold unsaturated side chains. Due to the presence of phenolic groups, vitamin E is easily oxidized and these derivatives are effective antioxidants. (Munne-Bosch and Falk, 2004; **Figure 5**). Among these derivatives, α-tocopherol has the highest biological activity for human health (Traber, 2007). Thus, the goal of vitamin E biofortification is to increase the contents of vitamin E and to convert all the other types into α-tocopherol.

Mostly, approaches to enhance vitamin E are to increase the activity of the enzymes in each step of the synthesis, including *p*-hydroxyphenylpyruvate dioxygenase (HPPD; Farre et al., 2012), homogentisate phytyltransferase (HPT1/VTE2; Seo et al., 2011), homogentisate geranylgeranyl transferase (HGGT; Cahoon et al., 2003), homogentisate solanesyltransferase (HST; Sadre et al., 2006), 2-methyl-6-phytyl-benzoquinol methyltransferase (MBPQ-MT/VTE3; Sattler et al., 2004; Tang et al., 2016), tocopherol cyclase (TC/VTE1; Kumar et al., 2005; Yabuta et al., 2013), and γ-tocopherol methyltransferase (γ-TMT/VTE4; Van Eenennaam et al., 2003; Cho et al., 2005; Tavva et al., 2007; Lee et al., 2008; Ghimire et al., 2011; Yabuta et al., 2013; Zhang et al., 2013; **Figure 5**).

Among the trials mentioned above, either a single gene or multiple genes in combination have been adopted. For example, when the barley *HGGT* gene is transformed into maize, the total tocotrienols and tocopherols increase around six folds (around 800 nmol g^-1^) in the transgenic seeds (Cahoon et al., 2003). Barley *HGGT* gene also improves all-trans β-carotene stability in transgenic sorghum events containing *AtDXS, PSY1*, and *CRTI* (Che et al., 2016). The most commonly used single-gene approach is the overexpression of γ-TMT, which results in an increased proportion of α-tocopherol among the tocopherols, such as in canola (7.0-fold increase to over 70% of total; Van Eenennaam et al., 2003), lettuce (over 2-fold increase to 99% of total; Sattler et al., 2004; Cho et al., 2005), *Perilla frutescens* (1.8-fold increase to 99% of total with the CaMV 35S promoter, Ghimire et al., 2011; 26.6-fold increase to 75% of total with a seed-specific Vicillin promoter, Lee et al., 2008), and soybean(7.0-fold increase to 75% of total, Sattler et al., 2004; 10.4-fold increase to 88% of total,10.4 folds, Tavva et al., 2007) Compared with the single gene, the overexpression of multiple genes involved in vitamin E biosynthesis usually results in more massive accumulation of tocotrienols, and the total vitamin E activity is dramatically increased (Collakova and DellaPenna, 2003; Van Eenennaam et al., 2003; Sattler et al., 2004). For example, the combination of HPT and γ-TMT results in a 12-fold increase of vitamin E activity up to 47.4 mg α-tocopherol 100 g^-1^ tissue in canola seeds (Collakova and DellaPenna, 2003); the combination of MT and γ-TMT results in a 5-fold increase in soybean (Van Eenennaam et al., 2003; Sattler et al., 2004); and the combination of *tyrA* (*HPT* in yeast), *Arabidopsis* HPPD and HPT results in an 11-fold increase in soybean (Karunanandaa et al., 2005). Besides, modulation of the activity of TC and MT also altered the α-/γ- tocopherol ratio in plants (Van Eenennaam et al., 2003; Kumar et al., 2005). Unfortunately, similar approaches do not bring about expected effects in maize and potato. For example, overexpression of *Arabidopsis* HPPD and MT results in an only 3-fold increase in γ-tocopherol in maize kernels, and other tocopherol isomers are undetectable (Naqvi et al., 2011). Similarly, constitutive overexpression of *Arabidopsis* HPPD or HPT does not affect the tocopherol composition in potato tubers (Crowell et al., 2008).

Genome-wide association study is also used to identify the natural allelic variations controlling vitamin E. Two insertion/deletions within *VTE4*, and a single nucleotide polymorphism (SNP) located 85 kb upstream of this gene are found to be significantly associated with α-tocopherol contents in maize kernels (Li Q. et al., 2012), and three genes, *VTE1, HGGT1* and a prephenate dehydratase paralog, are also found to modestly contribute to tocotrienol variations in maize (Lipka et al., 2013). In tomato, a short interspersed nuclear elements (SINE) retrotransposon located in the promoter region of *VTE3* is identified to be responsible for vitamin E accumulation in fruits (Quadrana et al., 2014). The above findings suggest the existence of as-yet-unknown candidate genes for use in vitamin E biofortification.

## Conclusion and Future Prospects

Promotion of nutrition-sensitive agriculture and food-based strategies can solve micronutrient malnutrition (FAO, 2014). A recent study of vitamin intakes in developed countries—including Germany, the UK, the Netherlands and the United States—reveals that although inter-country differences exist, intakes of several vitamins are below the recommended levels in a significant part of the population even in these countries. Moreover, there is a gap between vitamin intake and requirements for a significant proportion of the population, despite the availability of diverse foodstuffs (Troesch et al., 2012).

Two general questions must be addressed during the design of micronutrient-enriched crops: (1) can breeding increase the micronutrient density in staple foods to reach target levels that will have a measurable and significant impact on nutritional status, and (2) will the extra nutrients bred into food crops be bioavailable and absorbed at a sufficient level to improve micronutrient status when consumed under controlled conditions (Bouis et al., 2013)? Therefore, one must take into consideration the breeding targets, target nutrients, and fortification criteria after accounting for loss during storage, milling, processing and cooking, as well as the limited bioavailability (FAO/WHO, 2001; Hotz and McClafferty, 2007). Several reports in maize, tomato and lettuce have demonstrated the potential of biofortification in alleviating vitamin deficiencies in human, by the means of ensuring an adequate uptake of nutrients (such as β-carotene and folates) from biofortified foods without processing and cooking (Tang et al., 2012; Castorena-Torres et al., 2014; Kiekens et al., 2015). For example, after cooking, the nutrients in biofortified crops retained equivalent bioavailability as synthetic compounds: the β-carotene in Golden Rice was considered as effective as that in oil in terms of vitamin A supply to children (Tang et al., 2012), and the natural folates from biofortified tomato or rice had the potential to improve folate status in human (Castorena-Torres et al., 2014; Kiekens et al., 2015).

An integrated understanding of the genetic networks and the biochemical and molecular processes that control the accumulation of target compounds in crops is required (Bhullar and Gruissem, 2013), which, to our understanding, can be achieved by employing novel technologies, such as isotope labeling-based metabolomics, metabolite-macromolecule interactions and metabolite–micromolecule interactions. Furthermore, various approaches must be used to achieve preferential accumulation in the edible parts of crops, of minerals and/or vitamins or prebiotics (non-digestible carbohydrates) and reduce the antinutrient compounds, such as phytates and polyphenolics (Hurrell and Egli, 2010).

Human nutrition research is now focused on personalized approaches. It is also conceivable that personalized plant breeding would be a future prospect to meet the nutritional needs of individuals; this will require the development of new-generation gene-sequencing technologies and large data-processing systems (Crotti et al., 2017). To this end, the following are required: (1) analysis of the whole genome of crops to understand the genetic basis of nutritional traits and to resolve the nutrition-relating haplotypes; (2) large-scale mining of genes related to nutrition and health-related phenotypes in natural germplasm resources combining metabolome, genomics, and GWAS to integrate the elite allelic variations in crops; (3) application of metabolomics to identify intermediate metabolites and the rate-limiting steps of metabolic pathways; and 4) design and creation of novel metabolic pathways that produce nutrition- and health-related nutrients. Taking all together, we can foresee that a successful manipulation of metabolism in food crops will make substantial contributions to human health by improving the nutrition and wellbeing of the world population, especially the impoverished people.

## Author Contributions

LJ and CZ designed and wrote the paper. WW and TL wrote the paper.

## Conflict of Interest Statement

The authors declare that the research was conducted in the absence of any commercial or financial relationships that could be construed as a potential conflict of interest.

## Footnotes

1 http://ndb.nal.usda.gov/ndb/nutrients/index

2 http://www.nap.edu/catalog/11537.html

## Abbreviations

γ-TMT: γ-tocopherol methyltransferase
CRTB: Bacterial phytoene synthase
CTPCHI: GTP cyclohydrolase I
FAO: Food and Agriculture Organization of the United Nations
HGGT: homogentisate geranylgeranyl transferase
HPPD: p-Hydroxyphenylpyruvate dioxygenase
HPT: homogentisate phytyltransferase
HYDB: β-carotene hydroxylase
PSY: phytoene synthase
RDI: recommended daily intake
WHO: World Health Organization
